# Purinergic Signaling, HIF, and ROS Interactions in Myocardial I/R Injury: Therapeutic Potential and Future Prospective

**DOI:** 10.3390/cells15080682

**Published:** 2026-04-13

**Authors:** Manish Kumar Singh, Hyeong Rok Yun, Jyotsna Ranbhise, Sunhee Han, Hanjoon Seo, Seung Geun Yeo, Fu-Shi Quan, Sung Soo Kim, Insug Kang

**Affiliations:** 1 Department of Biochemistry and Molecular Biology, School of Medicine, Kyung Hee University, Seoul 02447, Republic of Korea; manishbiochem@gmail.com (M.K.S.);; 2 Biomedical Science Institute, Kyung Hee University, Seoul 02447, Republic of Korea; 3 Department of Biomedical Science, Graduate School, Kyung Hee University, Seoul 02447, Republic of Korea; 4 Department of Otorhinolaryngology—Head and Neck Surgery, Kyung Hee University Medical Center, College of Medicine, Kyung Hee University, Seoul 02453, Republic of Korea; 5 Department of Medical Zoology, School of Medicine, Kyung Hee University, Seoul 02447, Republic of Korea; 6 Medical Research Center for Bioreaction to Reactive Oxygen Species and Biomedical Science Institute, School of Medicine, Kyung Hee University, Seoul 02447, Republic of Korea

**Keywords:** adenosine, cAMP, ischemic, myocardial, mitochondrial, reperfusion

## Abstract

**Highlights:**

**What are the main findings?**

- Purinergic signaling acts as a primary regulator of inflammatory responses and is associated with ischemic tissue injury.
- Hypoxia-inducible factor (HIF) and reactive oxygen species (ROS) exhibit significant crosstalk with adenosine signaling during myocardial infarction (MI), myocardial ischemic/reperfusion injury (MIRI), and myocarditis.

**What are the implications of the main findings?**

- The synergistic regulation of the adenosine receptor and HIF signaling represents a potent therapeutic target.
- Pharmacological interventions that stabilize HIFs or selectively activate adenosine receptors offer a promising strategy for mitigating myocardial damage and improving clinical outcomes in patients with acute myocardial infarction (AMI).

**Abstract:**

Purinergic signaling plays a critical role in several inflammatory diseases, including acute lung injury, inflammatory bowel disease, coronary artery diseases, and various cancers. Purine and its derivatives, specifically adenosine and ATP, exhibit a critical regulatory axis that bridges platelet activation, vascular thrombosis, and sterile inflammation. Myocardial infarction (MI) initiates a complex pathophysiological cascade characterized by profound hypoxia, inflammation response, reduced coronary blood flow, and increased oxidative stress, which leads to myocardial cell death and apoptosis. Reperfusion therapy remains a primary strategy for restoring coronary blood flow and maximally limiting infarct size; increased infarct size further exacerbates ischemic injury, making it myocardial ischemic/reperfusion injury (MIRI). In this review, we delineate the mechanistic “triad axis”, comprising adenosine signaling, hypoxia-inducible factor (HIF) stabilization, and reactive oxygen species (ROS) homeostasis; this axis serves as a pivotal determinant of cardiomyocyte death during MIRI. We further examine the cell-specific roles of adenosine signaling in modulating immune cell infiltration and function within the ischemic milieu. Finally, we highlight the emerging role of mitochondrial ROS (mtROS) and HIF-dependent signaling in circadian regulation, suggesting that the chronotherapeutic approaches targeting these pathways may offer transformative opportunities for the treatment of ischemic heart disease (IHD).

## 1. Introduction

Myocardial infarction (MI) is a pathological condition characterized by acute myocardial injury resulting from prolonged ischemia [1]. Studies have demonstrated key clinical and cellular characteristics, including mitochondrial abnormalities and myocardial cell death due to apoptosis under sustained ischemic conditions. Prolonged ischemia leads to inadequate oxygen supply and is associated with a dynamic rise and/or fall in cardiac troponin (cTn) levels > 99th percentile of upper reference limit (URL), a defining criterion of AMI. Further, depending on clinical features such as cTn levels, ECG, angiographic data, or other imaging evidence, MI is classified into five subtypes (Types 1–5) [1,2,3]. Type 1 MI is usually caused by ischemia that results from acute coronary occlusion following the rupture of an atherosclerotic plaque [4]. In contrast, Type 2 MI results from ischemic imbalance from conditions unrelated to coronary artery diseases, including anemia, arrhythmia, hypotension, and hypertension [3]. Type 3 MI is reserved for patients who experience sudden cardiac death with symptoms of MI or ventricular fibrillation or through autopsy confirmation. Type 4a is classified based on cTn values > 5 times the 99th percentile URL in patients with normal baseline values. Additionally, Type 4b and Type 4c MI, as documented by angiography or autopsy, use the same criteria used for Type 1 MI. Type 5 MI is related to coronary artery bypass grafting (CABG) and requires cTn elevation > 10 times the 99th percentile URL (Table 1) [1]. Ischemia–reperfusion injury (IRI) frequently occurs in patients presenting with an acute ST segment elevation myocardial infarction (STEMI) in whom early restoration of blood flow (i.e., reperfusion therapy) represents the most effective therapeutic strategy to reduce ischemic injury and limit infarct size. Reperfusion is achieved primarily either through thrombolytic therapy or primary percutaneous coronary intervention (PCI) [5]. Alternatively, bypass surgery and PCI are widely used to eliminate vascular obstruction and restore normal vascular perfusion, which significantly reduces AMI-associated mortality [6]. Substantial studies indicate that extracellular adenosine and adenosine-mediated signaling pathways provide considerable cardioprotective effects during MIRI [7], underscoring their potential therapeutic relevance in the management of AMI.

### 1.1. Purinergic Signaling in Inflammatory Response

**Figure 1:**
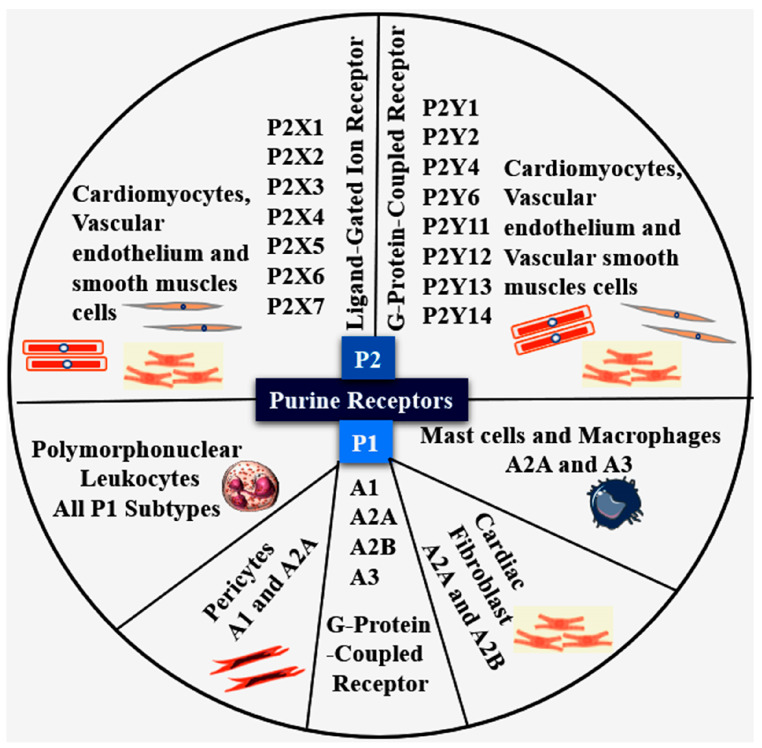
Schematic illustration of various purine receptors and their expression patterns and sub-cellular localization in cardiomyocytes and cardiac-infiltrating immune cells. Specifically, myeloid cells such as neutrophils, macrophages, and T lymphocytes express distinct purinergic receptor subsets that modulate chemotaxis, cytokine release, inflammasome activation, and the resolution of inflammation. The spatial distribution of these receptors across cardiac and immune compartments underscores their coordinated role in shaping inflammatory signaling, myocardial injury, and tissue repair following ischemic stress.

Purinergic signaling primarily involves extracellular purines and their derivatives, most notably adenosine, ATP, and ADP, acting through specific purinergic receptors. Accumulating evidence highlights its critical role in a broad range of human diseases such as acute lung injury, rheumatic disorders, inflammatory bowel disease, coronary artery diseases, and various cancers, underscoring its central importance in human pathophysiology. Notably, purinergic signaling constitutes a key regulatory axis linking platelet activation, vascular thrombosis, and inflammation [8]. It comprises purine nucleoside adenosine, which is generated from ATP and ADP via the coordinated action of ectonucleotidases CD39 and CD73 expressed on the cell surface; these also express the receptors that mediate purinergic signaling. The concept of extracellular purinergic signaling was first proposed by Geoffrey Burnstock in 1972 [9]. Among purine mediators, ATP and ADP play pivotal roles in platelet activation and aggregation. Initially, purine receptors are broadly classified into two families: P1 and P2. The P1 receptor family comprises four G-protein-coupled receptor (GPCR) subtypes (A1, A2A, A2B, and A3), which primarily regulate intracellular levels of cyclic adenosine monophosphate (cAMP). In contrast, the P2 receptor family is subdivided into seven ligand-gated ion channel (P2X1–P2X7) and eight GPCR subtypes (P2Y1, P2Y2, P2Y4, P2Y6, P2Y11, P2Y12, P2Y13, and P2Y14). A1 and A3 receptors negatively regulate adenylyl cyclase through coupling to Gi/o proteins, whereas A2A and A2B receptors positively regulate adenylyl cyclase via Gs proteins. Notably, the A2B receptor is also capable of coupling to Gq proteins [10]. During ischemic injury, intracellular ATP is released into the extracellular space and subsequently degraded into ADP, AMP, and adenosine. These endogenous ligands activate purinergic receptors expressed on cardiovascular tissues and circulating blood cells (Figure 1), thereby contributing to the complex pathophysiological mechanisms underlying MIRI [11].

Experimental studies have demonstrated that ADP induces platelet aggregation through the coordinated activation of P2Y1 and P2Y12, and the GPCRs [12]. Under ischemia and reperfusion conditions, extracellular ATP serves as a primary danger-associated molecular pattern (DAMP), promoting phagocyte recruitment [13] and stimulating proinflammatory cytokine such as IL-18 release through the activation of NLRP3 inflammasome, thereby driving sterile inflammatory response [14]. Conversely, accelerating the enzymatic conversion of ATP to adenosine has been shown to attenuate ischemic reperfusion (I/R)-induced tissue injury, highlighting the context-dependent protective role of the purinergic signaling network in sterile inflammation.

Both endogenous A1 receptor and exogenous A3 receptor activation confer protection against vascular injury and post-ischemic dysfunction [15], whereas A2A and A2B receptor subtypes exert cardioprotective effects primarily through metabolic regulation [16]. Mechanistically, A1 receptor signaling suppresses necrosis and apoptosis by modulating Bcl-2/Bax and caspase-3 activity, resulting in reduced infarct size during MIRI [17,18]. In contrast, P2 receptors such as P2X7, P2Y2, and P2Y11 receptors regulate cardiac cell death through Bcl-2 and caspase-8 dependent pathways [19]. Platelet aggregation contributes to MI by promoting microthrombus formation within small cardiac vessels and capillaries, triggering the release of potent vasoconstrictors, and the secretion of proinflammatory cytokines [20]. Study identified three P2 receptor subtypes in platelets, particularly P2Y1 and P2Y12 on ADP, and P2X1 on ATP, that contribute to this process. Consequently, targeting platelets via P2 inhibitors, particularly P2Y12 receptor inhibitors such as prasugrel [21], cangrelor [22], and ticagrelor [23], has demonstrated significant therapeutic benefits in MI that extend beyond their established anti-thrombotic effects. Notably, in vivo evidences further supports the cardioprotective potential of ticagrelor pretreatment, which has been shown to reduce infarct size, potentially through enhancement of platelet-derived adenosine signaling in the context of remote ischemic conditioning (RIC) [24].

Recent studies have demonstrated that exogenous adenosine induces interleukin-6 (IL-6) production in cardiac fibroblasts through GPCR signaling. The A2B receptor has been coupled to different signaling pathways, including the Gs-cAMP-PKA pathway and the Gq/G11-PLC-PKC pathway [25]. In murine cardiac fibroblast, treatment with the stable adenosine analog, adenosine-5′-N-ethyluronamide (NECA), stimulates IL-6 secretion via an A2B receptor-dependent protein kinase C, such as PKCδ-P38 mitogen-activated protein kinase (MAPK) signaling pathway [26]. Subsequently, evidence has extended these findings from exogenous stimulation models to physiological paracrine signaling, revealing that T cell-derived adenosine serves as a primary driver of fibroblast IL-6 production in the infarcted heart via A2B-Gq coupling. However, whether p38 MAPK signaling is directly required for T cell-initiated A2B-Gq activation remains unclear and warrants further investigation.

### 1.2. HIF Signaling and Inflammatory Response

HIFs are ubiquitously expressed heterodimeric transcription factors consisting of two forms: oxygen-regulated α-subunit (HIF1α) and a constitutively expressed β-subunit (HIF1β). HIF1α consists three isoforms HIF-1α, HIF-2α, and HIF-3α [27]. Among these, HIF-1α, identified by Semenza and colleagues in 1991, is the most extensively studied and plays a central role in coordinating the cellular response to hypoxia [27,28]. The activation of the HIF pathway and transcription depends on the cellular oxygen level. Additionally, HIF-prolyl hydroxylase (HIF-PHDs) activity is oxygen-dependent, linked with hydroxylation of HIF-1α by two conserved proline residues at pro402 and pro564. The HIF-PHDs consist of three different isoforms, including PHD1 (EGLN2), PHD2 (EGLN1), and PHD3 (EGLN3) [29]. Under normal oxygen levels, prolyl hydroxylation promotes the binding of the Von Hippel–Lindau (pVHL) gene product E3 ubiquitin ligase complex to the HIF-1α and promotes its proteasomal degradation [7]. Conversely, under hypoxia conditions (limiting oxygen availability), the hydroxylation activity of PHD proteins is inhibited, leading to stabilization of HIF-1α and subsequent activation of its target gene expression [30]. Another level of control lies with asparaginyl hydroxylase factor inhibiting HIF (FIH), which is also oxygen-sensitive and iron-dependent, and facilitates the recruitment of transcriptional cofactors, such as p300, that are necessary for HIF-mediated gene transcription [31,32]. Thereby, both HIF-PHDs and FIH serve important roles as oxygen sensors by independent, oxygen-dependent activity in regulating HIFs activity [33].

Activation of HIF-targeted genes enables the cellular metabolism and functioning of several cell types, such as neutrophils and other myeloid cells, such as monocytes, in human sepsis [34,35]. Neutrophils confined to blood vessels pass through small postcapillary veins into the extravascular tissue at the site of infection (or injury). The activated vascular endothelium allows selective extravasation of neutrophils, which are activated upon arrival at the site of the affected tissue and then from substrates such as ROS and reactive nitric oxide (NO), which are effectors that will discriminately damage pathogens and the human body, resulting in damage to human tissues [36]. HIF-1α can indirectly enhance inflammatory responses by promoting the production of inflammatory cytokines, while conversely, under certain conditions, it can also suppress excessive inflammatory responses by inhibiting the activation of NF-κB [37,38,39].

HIF signaling has been implicated in a wide range of inflammatory disorders, including systemic lupus erythematosus (SLE), rheumatoid arthritis (RA), systemic sclerosis (SS), and psoriasis [33]. Accordingly, targeting HIF-1α has emerged as a promising strategy for alleviating inflammatory diseases. Studies have demonstrated that both low oxygen and inflammation promote the release of extracellular ATP and ADP [8], which are subsequently metabolized to adenosine at the cell surface through the coordinated actions of ectonucleoside triphosphate diphosphohydrolase-1 (CD39) and ecto-5′-nucleotidase (CD73) under low oxygen conditions. Notably, CD39 expression is transcriptionally regulated by the Sp1 transcriptional factor, and its induction has demonstrated protective effects in experimental models of hypoxia and ischemia [40]. Consistent with these findings, studies in CD39-deficient mice have revealed that neutrophil-derived extracellular adenosine represents an immediate anti-inflammatory response to hypoxic stress [41]. Beyond CD73, adenosine receptors, particularly A2AAR and A2BAR, are themselves transcriptional targets of HIFs. Notably, the A2AAR and A2BAR expression are differentially regulated by HIFs, with A2AAR primarily controlled by HIF-2α and A2BAR by HIF-1α [42,43]. Under hypoxic conditions, A2BAR expression is significantly upregulated [41,43,44]. In contrast, A2AAR transcription is specifically induced in pulmonary endothelial cells at low oxygen levels via direct binding of HIF-2α to its regulatory regions [42]. The transgenic mice with genetic deletion of either CD73 or A2BAR experience more severe tissue injury following ischemia [45,46]. Conversely, pharmacological stabilization of HIFs using dimethyloxalylglycine (DMOG) fails to confer protection in CD73- or A2BAR-deficient mice, underscoring the essential role of adenosine signaling axis in HIF-mediated tissue protection during ischemia [47].

HIFs have also been implicated in attenuating the termination of adenosine signaling. ENT1 and ENT2 are the main adenosine transporters that orchestrate the movement of adenosine from the extracellular cell surface to the intracellular space in vascular endothelial and epithelial cells. Under hypoxic conditions, adenosine kinase (AK) activity is reduced, diverting myocardial adenosine away from the salvage pathway and toward venous release [48]. Experimental gain- and loss-of-function studies demonstrate that HIF-1α suppresses AK transcription, implicating this mechanism in hypoxia-induced vascular leakage observed in conditions such as sepsis or acute lung injury [49]. Collectively, these findings highlight a central role for HIF-1α in coordinating the transcriptional regulation of multiple components of the adenosine pathway under hypoxic conditions.

**Figure 2:**
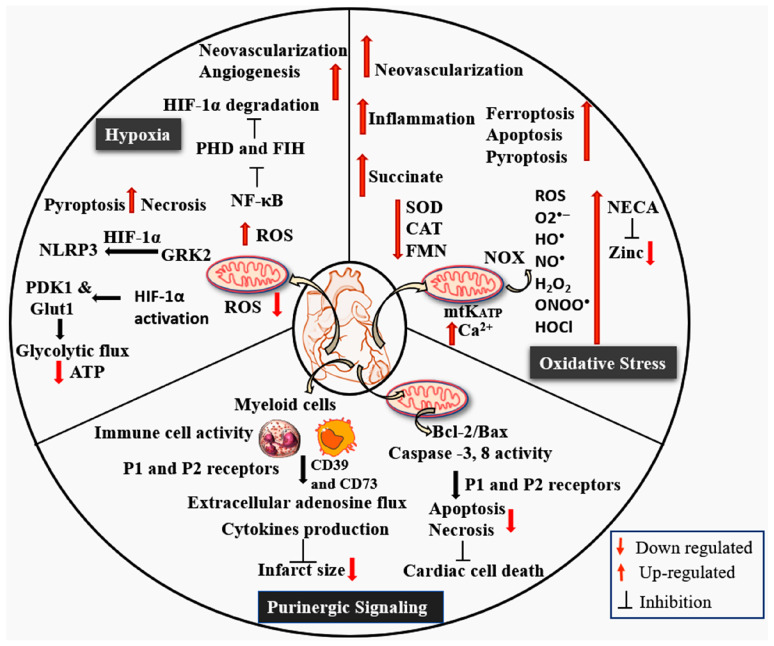
The image illustrates the interconnected molecular pathways driving MI and subsequent cardiac injury. Purinergic signaling, hypoxia, and the production of ROS represent central mechanisms in MI progression. During ischemia, extracellular adenosine is released, which activates cell-surface purinergic receptors, amplifying immune cell recruitment and the production of cytokines. Hypoxia enhances oxidative stress and ROS accumulation, triggering cell death programs including ferroptosis, apoptosis, and pyroptosis, thereby exacerbating cardiomyocyte injury. Additionally, mitochondrial oxidative stress leads to succinate accumulation, intracellular calcium overload, and the opening of ATP-sensitive potassium (K_ATP_) channels, further disrupting bioenergetic balance. Moreover, purinergic signaling plays a role in modulating mitochondrial-dependent cell death pathways, including apoptosis and necrosis.

Recently, alternative HIF-dependent pathways linked to circadian regulation have been identified as critical determinants of diurnal variation in acute myocardial infarction injury. Cellular and genetic studies in mice have revealed that BMAL1, a core transcription factor, regulates circadian-dependent myocardial injury [50,51] by forming a transcriptionally active heterodimer with a non-canonical partner-HIF-2α in a time-of-day-dependent manner [52,53]. BMAL1 enhances the hypoxic response by stabilizing HIF-2α and enhancing its transcriptional activity under the circadian hypoxic response. Notably, amphiregulin (AREG), a downstream target for BMAL1, exhibits a critical role in regulating daytime variations in myocardial injury and infarct size (Figure 2). Pharmacological targeting of the BMAL1-HIF-2α-AREG axis confers cardioprotection, with maximal efficacy achieved when treatment is aligned with the pathway’s circadian phase. These findings highlighted that chronotherapeutic strategies are needed to optimize the HIF-targeted interventions. The combined modulation of BMAL1 and HIF-2α represents an alternative signaling cascade that may offer novel opportunities for the treatment of IHD.

### 1.3. Mitochondrial ROS and Inflammation Response

Mitochondria are the primary source of ROS in cardiomyocytes. Cardiomyocytes are specialized cells in the heart muscles and consist of a high number of mitochondria. In cardiomyocytes, ROS is produced by the oxidative respiratory chain reaction, which supports the development and maturation of cardiomyocytes under normal physiological conditions. In contrast, dysregulated ROS accumulation leads to various cardiovascular diseases, such as myocardial hypertrophy, hyperlipidemia, myocardial ischemia/reperfusion injury, arrhythmias, and diabetic cardiomyopathy. Free radicals such as superoxide anion (O2^•−^), hydroxyl radical (HO^•^), nitric oxide (NO^•^), and lipid radicals with unpaired electron pairs, while hydrogen peroxide (H_2_O_2_), peroxynitrite (ONOO^−^), and hypochlorous acid (HOCL) are non-free radicals. Mitochondrial NADPH oxidase (Nox) is the major source of H_2_O_2_ and superoxide produced by different isoforms of Nox family proteins. Nox, a multi-transmembrane protein, comprises seven isoforms, namely, Nox1-Nox5, Duox1, and Duox2. Nox1, Nox2, and Nox4 are expressed in endothelial cells, vascular smooth muscle cells, fibroblasts, and cardiomyocytes [54,55,56]. Superoxide and oxidative stress, driven by dysregulation of ROS homeostasis, significantly contribute to cardiomyocyte death during myocardial I/R injury, as well as doxorubicin-induced cardiomyopathy. This stress primarily induces ferroptosis, a regulated cell death process crucial to the progression of cardiovascular disease [57]. ROS also trigger pyroptosis, characterized by plasma membrane pore formation, cell swelling, osmotic lysis, and the release of proinflammatory mediators [58].

Reperfusion is essential for restoring blood flow following ischemia; however, it paradoxically triggers a rapid oxidative burst driven by mitochondrial dysfunction and ionic imbalance, which activates the xanthine oxidoreductase (XOR) system, leading to the substantial production of ROS [59]. XOR exists in two interconvertible forms: xanthine oxidase (XO) and xanthine dehydrogenase [60]. Notably, XO-derived ROS are generated within minutes of reperfusion, whereas neutrophil-driven ROS content, primarily mediated by NADPH oxidase, typically peaks several hours later [61]. In vivo studies have demonstrated that superoxide production during the early phase of I/R injury is markedly attenuated by allopurinol, a potent XO inhibitor [62], confirming XO as a primary vascular source of ROS in the rat forebrain. Similarly, febuxostat has shown comparable efficacy in preclinical I/R models, although its clinical utility remains under investigation. Emerging evidence underscores the therapeutic potential of XO inhibition in reducing oxidative stress across multiple organ systems, including the heart, kidneys, and brain [63]. Importantly, the protective effect of allopurinol is not associated with disruption of the Nrf2 signaling pathway, suggesting that XO inhibition can be effectively combined with immune modulatory and anti-inflammatory strategies to achieve synergistic benefits [64]. Collectively, these findings highlight XOR as a critical therapeutic target, where its inhibition may suppress ROS overproduction, preserve endogenous antioxidant defenses such as catalase activity, and ultimately mitigate myocardial injury in cardiac I/R models [63].

Additionally, disruption of circadian rhythms exacerbates oxidative stress in cardiomyocytes by reducing antioxidant enzymes such as superoxide dismutase (SOD) and catalase (CAT), while increasing mtROS production [65]. Mitochondrial complex I is a key source of ROS, driven by NADH/NAD^+^ redox imbalance and succinate-driven reverse electron transfer [66]. An upregulated NADH/NAD^+^ ratio increases fully reduced flavin mononucleotide (FMN) levels, facilitating electron transfer to oxygen and producing O2^•−^ ions. The flow of electrons into complex I can also be reversed when the coenzyme Q pool is reduced, further promoting mtROS production (Figure 2) [67]. Succinate plays a role in inducing mtROS during reperfusion, which contributes to IR injury in various tissues. The ischemic accumulation of succinate may also be significant due to its involvement in inflammatory and hypoxic signaling pathways [68]. Consequently, succinate may contribute both to the acute pathogenesis of IR injury via mtROS production and, following its release, to the subsequent activation of inflammatory responses and neovascularization [69].

Overall, mtROS generation is tightly linked to mitochondrial membrane potential (Δp), where mechanisms such as proton leakage can reduce ROS levels. Notably, downregulation of uncoupling protein 2 raises ROS in doxorubicin-treated hearts, a change reversible by matrine [70]. Additionally, superoxide can also arise from the autoxidation of FADH2 or its semiquinone intermediate [71]. Substantial ROS activates NF-κB and inhibits PHD and HIF asparaginyl hydroxylase (FIH) enzymes, preventing HIF-1α degradation and facilitating its nuclear translocation [72,73]. Simultaneously, ROS- induced G protein-coupled receptor kinase 2 (GRK2) interacts with HIF-1α to derive NLRP3 transcription, triggering caspase-1-mediated maturation of IL-1β and IL-18 and subsequently pyroptosis [74]. Conversely, low ROS or attenuated HIF-1α stabilization downregulates pyruvate dehydrogenase kinase 1 (PDK1) and glucose transporter 1 (GLUT1), impairing glycolytic flux and ATP production [75].

Enhanced ROS triggers the inflammation that initiates NLRP3 inflammasome activation and subsequent pyroptosis in cardiomyocytes [76]. The NLRP3 inflammasome is currently the most well-characterized can lead to the production of ROS at the mitochondrial level in different cellular models. Inflammasomes are multiprotein complexes composed of pattern-recognition receptors, including NLRP1, NLRP3, NLRC4, AIM2, or pyrin, together with inflammatory caspases, with or without the adaptor protein apoptosis-associated speck-like protein (ASC) [77]. Among them, the NLRP3 inflammasome is activated by a wide range of pathogen-associated molecular patterns and damage-associated molecular patterns, and consists of NLRP3, ASC, procaspase-1 and pro-interleukin-1β (IL-1β) [76]. Upon activation, NLRP3 recruits and promotes the autocatalytic cleavage of pro-caspase-1 into its active p20 and p10 subunits. Active caspase-1 subsequently processes pro-IL-1β and pro–interleukin-18 into their mature forms and cleaves gasdermin D (GSDMD), generating an N-terminal fragment that forms membrane pores, leading to pyroptotic cell death and cytokine release [78]. In hypoxia/reperfusion models of adult rat cardiomyocytes, induced pyroptosis has been shown to promote the activation of the NLRP3/caspase-1 axis, accompanied by calcium overload, which further drives inflammasome assembly [79,80]. A few microRNAs have emerged as critical regulators of MIRI, modulating NLRP3 activation and downstream inflammatory responses [81]. Among these, miR-223-3p negatively regulates the NLRP3 inflammasome by targeting the 3′-untranslated region (UTR)-binding sites of NLRP3 mRNA in myeloid cells [82]. Accordingly, suppression of miR-223-3p enhances NLRP3 activation and promotes the release of proinflammatory cytokines IL-1β and IL-18 [82].

Both experimental and clinical evidence supports NLRP3 inhibition as a promising strategy to attenuate myocardial injury, preserve cardiac function, and limit adverse remodeling. For instance, INF4E, a small-molecule inhibitor, has been shown to suppress NLRP3 activation and improve post-ischemic cardiac function [83]. Similarly, the 1,3,4-oxadiazol-2-one derivative compound 5, INF200, significantly reduces I/R induced NLRP3 activation, inflammation, and oxidative stress [84], while also ameliorating obesity-associated cardiometabolic dysfunction. Alternatively, targeting downstream effectors has also yielded beneficial effects on I/R injury. For instance, inhibition of IL-1β via anakinra and canakinumab reduces C-reactive protein levels and is associated with improved exercise capacity and quality of life [85]. Collectively, these findings underscore the central role of the NLRP3 inflammasome in the pathogenesis of cardiovascular diseases, including diabetic cardiomyopathy, myocardial infarction, I/R injury [86], and myocarditis [87]. Nevertheless, the optimal timing and clinical integration of inflammasome-targeted therapies remain to be fully established.

## 2. Adenosine Receptors and Cellular Functions

Adenosine receptors (ARs) are present on many immune cells, such as neutrophils, monocytes, macrophages, dendritic cells, T cells, B cells, and NK cells, as well as on parenchymal cells, where they are involved in the regulation of inflammation [88,89]. Adenosine is a nucleoside that can be produced in the extracellular space through the degradation of ATP by CD39 and CD73, which are membrane-associated enzymes. Adenosine exerts its effects through the binding to P1, or itself signals through four receptor subtypes (A1, A2A, A2B, and A3) and through triggering intracellular signaling mechanisms that regulate the production of cAMP [10,90,91]. These receptors are coupled to either Gi (A1 and A3) or Gs (A2A and A2B) proteins that inhibit or activate cAMP production, respectively [10]. An induced level of cAMP triggers the release of IL-10, the inhibition of leukocyte infiltration, and proinflammatory cytokines [92]. Apart from norepinephrine, neurons of the SNS release other transmitters, including ATP.

**Figure 3:**
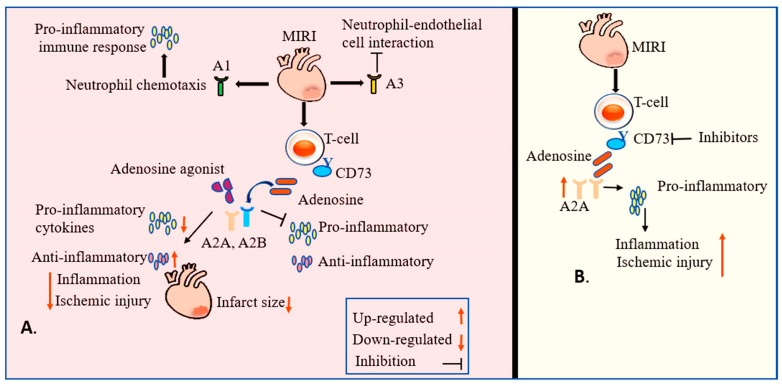
Schematic illustration of the interplay between adenosine signaling in modulating immune responses during MI. (**A**) Myeloid-derived immune cells, including T lymphocytes and neutrophils, sense fluctuations in extracellular adenosine concentration through purinergic receptors, leading to activation of inflammatory pathways and release of both proinflammatory and anti-inflammatory cytokines, resulting in exacerbation of ischemic injury. Pharmacological activation of adenosine receptors with selective agonists counteracts maladaptive immune activation, attenuates inflammatory signaling, limits infarct size, and reduces cardiomyocyte damage. (**B**) CD73-specific inhibition of adenosine and stimulation of inflammatory response result in increased ischemic injury.

Inadequate oxygen (hypoxia) results in impaired mitochondrial functions and reduced ATP production. ATP is quickly metabolized to adenosine, which is sensed by adenosine receptors. Also, adenosine can be directly transported to the intracellular compartment via equilibrative nucleoside transporters (ENTs), which are polytopic integral membrane proteins [8,93]. Within the intracellular compartment, adenosine is rapidly metabolized to inosine through adenosine deaminase (ADA) [94] or to AMP through adenosine kinase [49]. Adenosine receptor A2A (ADORA2A) has demonstrated a protective role in models of ischemia and reperfusion via inflammatory cell response; in contrast, A2B has shown cryoprotective effects via stabilizing HIF-1α and metabolic switch to glycolytic metabolism and Per2, a circadian rhythm protein, from ischemia [95]. Per2-deficient mice further exhibited a limited ability to utilize carbohydrates for aerobic glycolysis, which is attributed to HIF-1α stabilization in the ischemic heart. The A2B receptor may be important for maintaining the confirmation of the adenosine/A2A agonist binding site. Notably, A2B is also necessary for the assembly of the cAMP-signalosome downstream of A2A receptor activation [96]. In vivo studies have demonstrated that exogenous administration of 5′-(N-ethylcarboxamido) adenosine (NECA), an adenosine receptor agonist, increases vascular permeability and promotes polymorphonuclear neutrophil extravasation in CD73-deficient mice; these effects can be recapitulated by exogenous reconstitution with a soluble CD73-like nucleosidase [97]. However, long-term administration of beta 2-AR agonists exerts detrimental effects on cardiac function [98], thereby limiting their therapeutic applicability in conditions such as sarcopenia (Figure 3).

During MIRI, adenosine is released in large amounts and subsequently activated by A1AR and A3AR to prevent the damage from ischemic preconditioning [99]. The A3AR is expressed in various tissues and organs, including the testes, lungs, kidneys, heart, brain, and spleen; however, its expression varies among species [100]. Notably, A3AR is highly expressed in immune and inflammatory cells, particularly neutrophils, eosinophils, and mast cells, suggesting its crucial role in inflammatory response [101,102]. Recent evidence also demonstrated the crucial role of A3AR receptor in cardioprotection, similar to that activated by A1AR [103]. During ischemia, activation of A3AR exerts strong anti-inflammatory effects, limiting further damage to the myocardial tissues. Accumulating evidence indicates that A3AR agonists can reduce the infarct size by activating pro-survival signaling pathways, such as extracellular signal-regulated kinase (ERK1/2) and phosphoinositide 3-kinase (PI3K)/Akt pathways [104,105]. Despite its role in promoting growth in the heart, the genetic ablation of A3AR has been shown to reduce pathological cardiac remodeling characterized by hypertrophic growth and fibrotic changes [106]. Furthermore, A3AR signaling has been implicated in the modulation of intracellular calcium release in isolated human atrial myocytes [107], indicating its role in calcium homeostasis.

Several pharmacological A3AR agonists have demonstrated cardioprotective efficacy in preclinical models. For instance, CP-532903 (piclidenoson) has been shown to protect against I/R injury in mice via activation of sarcolemmal K_ATP_ channels [108], and similar effects have been observed in isolated adult mouse ventricular cardiomyocytes [109]. The activation of A3AR with 2-chloro-N6-(3-iodobenzyl) adenosine-5′-N methylcarboxamide (Cl-IB-MECA) has also been associated with enhanced pro-survival signaling pathways, resulting in reduced caspase-3 activity in rat hearts, which in turn results in reduced apoptosis and improved myocardial survival following I/R injury [104,110]. Additionally, recent evidence in animal models demonstrates that namodenoson and piclidenoson confer cardioprotection when administered during reperfusion, preventing I/R injury. Collectively, these findings indicate that A3AR agonists could be a promising therapeutic strategy for treating acute myocardial infarction [111]. Notably, its high dose has been associated with systemic hypertension in certain animal models, necessitating further investigation to establish optimal dosing, safety, and efficacy across species [112,113]. Currently, no A3AR agonist has been advanced to clinical trials specifically targeting cardiovascular disease, highlighting a critical gap between preclinical discovery and clinical application.

CD73 displays a pivotal role in the extracellular conversion of ATP to adenosine across the surface of diverse cell types [114]. CD73-derived adenosine on T cells is crucial for regulating chronic ventricular remodeling following transverse aortic constriction (TAC)-induced cardiac injury and may protect against maladaptive tissue remodeling [115]. Conversely, CD73 expression on B cells, natural killer cells, granulocytes, and monocytes remains lower and unchanged. Extracellular ATP activates the NLRP3 inflammasome via P2X7 receptor signaling, promoting inflammatory cytokines such as interleukin IL-1 β [116]. Clinical evidence underscores the importance of this pathway; loss-of-function mutations in the CD73-encoding gene NT5E cause a rare, severe, adult-onset disorder characterized by symptomatic arterial and joint calcifications in humans [117]. Despite these insights, the sexual dimorphism of the CD73/ adenosine axis during cardiac dysfunction and MIRI remains largely uncharacterized. Recent evidence from hypobaric hypoxia (HH) models demonstrates that the CD73 axis is sex-dependent; male mice showed more severe phenotypes than female mice following pharmacological inhibition of CD73 with adenosine 5′-(α, β-methylene) diphosphate (APCP) [118]. These observations suggest that the CD73/adenosine axis is a critical determinant of sex-specific inflammatory responses and represents a promising therapeutic target for mitigating proinflammatory injury in MIRI.

Quest et al. have shown enhanced P2X7 expression increases cardiac-infiltrating T cells, supporting the idea that T cell-derived ATP reduces the production of proinflammatory cytokines, thereby limiting proinflammatory response by paracrine and autocrine feedback mechanisms [119]. The degradation of ATP mediated by CD39 on activated T cells is derived from increased expression of the ectonucleotide pyrophosphatases ENPP1 and ENPP3, which hydrolyze ATP to AMP and pyrophosphate [120]. Increased CD73 expression reduces proinflammatory ATP and promotes the generation of anti-inflammatory adenosine [121]. The genetic deletion of either CD39 or CD73 is not lethal, indicating that extracellular phosphohydrolysis is dispensable under homeostatic conditions. Similarly, the viability of adenosine receptor-knockout mice suggests a degree of functional redundancy within the purinergic system under basal physiology.

In addition, CD38, a nicotinamide adenine dinucleotide nucleosidase, degrades extracellular NAD^+^ released from T cells via ENPP1 to AMP and adenosine [120,122]. CD73-deficient T cells produce enhanced secretion of Th17-associated cytokines (IL-6, IL-10, and IL-17), as well as increased production of IL-3, IL-13, MIP-1α, and MIP-1β [120]. Elevated IL-6 and IL-17 are strongly associated with congestive heart failure and enhanced cardiac fibrosis [123,124]. Although IL-10 and IL-13 possess anti-inflammatory properties, their increased expression inhibits the activation of monocytes and macrophages, which attenuates pressure overload-induced hypertrophic remodeling via STAT3-dependent inhibition of nuclear factor-κB (NF-κB) [125,126].

## 3. The ROS Connection with Purinergic Signaling in I/R Injury and Recovery

During ischemic reperfusion, ROS is markedly increased in the myocardium. Excessive ROS contributes to cardiomyocyte death and subsequent cardiac dysfunction [127]. Mitochondria are the major source of ROS, generating superoxide and/or H_2_O_2_ through the electron transport chain (ETC). Recent studies have demonstrated that reverse electron transport (RET) facilitates electron flow from reduced CoQH_2_ back to complex I, where NAD^+^ is reduced to NADH at the FMN site. This resulting accumulation of increased electron concentration at FMN promotes the reduction of molecular oxygen to superoxide [128]. During ischemia, succinate accumulates as an electron sink and, upon reperfusion, drives a burst of superoxide production from complex I via RET [129]. Additionally, mitochondrial complex I deficiency promotes glycolysis and increased mtROS production in macrophages; however, in the absence of an additional stimulus, this does not directly induce inflammation. Instead, this metabolic shift primes myeloid cells for prolonged inflammatory response, impairs effectocytosis, and delays the transition to tissue repair following MI. Notably, normalization of mtROS levels in the hearts of myeloid-specific complex 1-deficient mice reduces infarct rupture and improves survival after MI [130]. MI can lead to immediate necrosis of cardiac myocytes, triggering a robust inflammatory response. Neutrophils are the primary immune cells to accumulate in the infarcted myocardium, where they clear cellular debris and promote the recruitment of additional leukocytes such as LY-6C^hi^ monocytes and macrophages [131].

Increased oxidative stress also suppresses regulatory mechanisms that normally restrain inflammation, thereby impairing the resolution of the immune response. A key mechanism linked with this disruption of immune checkpoint pathways mediated by programmed cell death protein 1 (PD-1) and cytotoxic T-lymphocyte associated protein 4 (CTLA-4), resulting in persistent immune activation and progressive tissue injury [132]. CTLA-4 competes with CD28 for binding to B7 ligands (CD80/CD86), thereby limiting costimulatory signaling and suppressing T-cell activation [133]. PD-1 is expressed on activated T-cells, B-cells, and monocytes and plays a central role in immune regulation [134]. While CTLA-4 dampens IL-1-driven inflammatory responses, the PD-1 pathway is critical for modulating IL-6 signaling [135,136]. Oxidative stress further disrupts immune homeostasis by altering the balance between Treg/Th17 cells. Under physiological conditions, Treg suppresses excessive immune activation [137]. However, increased ROS and RNS favor Th17 differentiation through activation of STAT3 and RORγT, thereby enhancing inflammatory responses [138]. During ischemia, hypoxia induces HIF-1α stabilization, which activates proinflammatory gene expression and promotes anaerobic metabolism [139,140]. This metabolic shift causes mitochondrial damage and release of cytochrome c and mitochondrial DNA, which activate pattern recognition receptors and amplify inflammatory signaling. Upon reperfusion, the abrupt reintroduction of oxygen triggers a massive oxidative burst, further enhancing production of ROS [132]. This surge activates NF-κB signaling and stimulates the NLRP3 inflammasome, sustaining a feed-forward inflammatory cascade [141,142].

Hypoxia associated with MI results in HIF stabilization, and several studies suggest that alterations in purinergic signaling are linked to alterations in these events [143]. Adenosine signaling has been shown to exert protective effects in multiple forms of organ injury, including cardiac injury [144,145]. Importantly, the promoter region of the A2B adenosine receptor contains a hypoxia-response element (HRE), enabling binding of hypoxia-inducible transcription factors and resulting in increased A2B receptor expression under hypoxia or inflammatory conditions [146]. Notably, A2BR signaling has demonstrated significant cardioprotective effects during MIRI. For instance, ADORA2B-deficient mice abolish the protective effects of ischemic preconditioning and result in increased infarct size [45]. Moreover, the selective A2B agonist BAY 60-6583 effectively attenuates MI [147] during reperfusion and reduces lung injury induced by mechanical ventilation or LPS exposure [146]. Similar protective effects have been observed in inflammatory peritonitis, where sevoflurane stabilizes HIF transcription factors and enhances extracellular adenosine signaling through A2B adenosine receptor activation [148].

## 4. A Triad Crosslink Between Purinergic Signaling, HIF and ROS

### 4.1. Purinergic Modulation of HIF Stability

Purinergic signaling exerts both positive and negative regulatory effects on MIRI through the selective activation of adenosine receptors. A1 receptor activation stimulates neutrophil chemotaxis, thereby promoting a proinflammatory immune response during MIRI. Conversely, studies indicate that A3 adenosine receptor (A3AR) activation can attenuate MIRI by suppressing neutrophil-endothelial cell interactions, although it may also modulate specific proinflammatory pathways [149]. The A2 receptor subtypes, however, are considered the primary mediators of the inflammatory immune responses in I/R injury. Adenosine derived from T-cell CD73 acts on A2A and A2B receptors to modulate the secretion of proinflammatory and profibrotic cytokines [150]. Specifically, the activation of A2A and A2B receptors can inhibit the release of several proinflammatory cytokines, including TNF-α, INF-γ, IL-1α, IL-1β, IL-2, and IL-6. Furthermore, A2A receptor stimulation promotes the secretion of anti-inflammatory cytokines, such as IL-10 [151]. Consequently, targeting the A2A receptor through the exogenous administration of adenosine may regulate the infiltration of immune cells at the site of injury, thereby minimizing myocardial damage. Notably, endogenous ADO is critically required at the onset of reperfusion to trigger ischemic post-conditioning (post-C). A brief exposure to ADO antagonist has been shown to abrogate the cardioprotective effect of post-C, underscoring the essential role of endogenous ADO signaling in limiting the infarct size expansion [152].

T cell differentiation analysis revealed that CD73 does not directly influence T cell polarization, although the memory T cell marker CD44 has significantly increased, suggesting its critical role in regulating proinflammatory conditions [153]. The A2 adenosine receptor (A2AR) has been significantly upregulated on activated cardiac-infiltrating T cells and contributes to limiting inflammation; however, CD73 deficiency could limit its positive outcomes [119,154]. These observations highlight the therapeutic potential of selective A2AR agonists to attenuate immune-driven cardiac remodeling with limited adverse vasodilator responses [155]. Notably, A2AR signaling in cardiac fibroblasts also modulates collagen composition within scar tissue, further supporting its relevance in post-injury remodeling [156].

**Figure 4:**
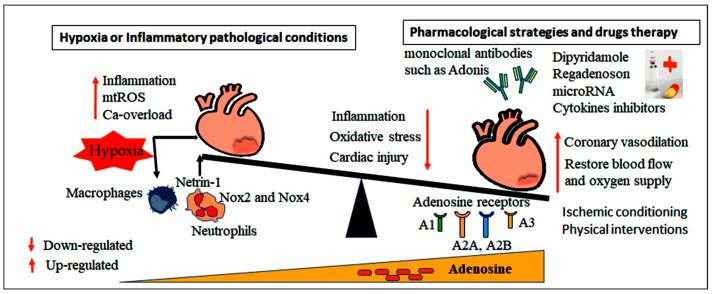
Schematic illustration depicting the integrated pharmacological strategies that target adenosine signaling and immune response during MIRI. Pharmacological agents and monoclonal antibodies modulate the availability of extracellular adenosine and its receptor signaling, thereby attenuating the inflammatory response induced by ischemia. Concurrently, the regulation of myeloid and lymphoid immune cell populations, including T-cells (Treg/Th1) and neutrophils, enhances the expression of netrin-1 and modulates the activity of NADPH oxidase (Nox), leading to reduced ROX generation. The combined effects of modulating adenosine signaling and immune cell reprogramming synergistically suppress inflammation, limit oxidative stress, and mitigate cardiomyocyte injury.

In addition to the basal level of ATP during I/R injury, the heart needs extra energy for maintaining cardiac function. During hypoxia, the glycolytic genes expression are induced by HIF-1α to maintain ATP production and prevent increases in ROS production [157]. HIF-1α inhibits fatty acid oxidation (FAO) through suppression of peroxisome proliferator-activated receptor-α (PPARα), a master regulator of FAO, in the heart. A study demonstrated the critical role of Nox2 and Nox4 in mediating ROS production and myocardial injury in response to I/R. Interestingly, a low level of ROS produced by either Nox2 or Nox4 is required for the heart to activate adaptive mechanisms, including regulation of HIF-1α and (PPARα) (Figure 4). This indicates that both Nox2 and Nox4 have pathological and adaptive roles in the heart during MIRI [158].

Ischemia and hypoxia cause the extracellular accumulation of adenosine primarily through the activation of CD39 and CD73, which catalyze the sequential degradation of ATP to adenosine, resulting in a significant extracellular flux of adenosine. During myocardial ischemia, adenosine concentrations can rise to higher than 20-fold, activating all adenosine receptor subtypes [159]. In reactive hyperemia, ATP release from endothelial cells via NO pathways initiates early vasodilation, which is further amplified as ATP degrades into adenosine (Figure 4) [160,161]. Accumulating evidence suggests that adenosine administration reduces the risk of major adverse cardiovascular events and heart failure, primarily through limiting infarct size and preserving cardiac function. However, its effect on all-cause mortality, cardiac death, thrombosis, and reinfarction appears unaffected [162]. In STEMI, pharmacological adenosine has demonstrated efficacy in mitigating the no-reflow phenomenon. It serves as a key component of multimodal reperfusion strategies aimed at improving microvascular function and enhancing post-PCI recovery [163].

### 4.2. Purinergic Modulation of Oxidative Stress Signaling

Adenosine exerts myocardial protective effects by reducing inflammation, oxidative injury, and metabolic demand during ischemic injury. Activation of A1R and A3R promotes myocardial protection and collateral circulation, especially A2AR and A2BR, which drive vascular dilation to improve oxygen delivery and ATP generation [164]. Furthermore, enhanced CD39 and CD73 activity, particularly on neutrophils, significantly increases extracellular adenosine production, reinforcing its accumulation during ischemia [165]. Hypoxia also upregulates A2AR expression by NF-κB and HIF-1α-dependent pathways, suppressing inflammation and immune responses [166]. Both A2AR and A2BR are critical for limiting ischemia–reperfusion injury and reducing myocardial oxygen demand, highlighting their therapeutic potential in myocardial infarction [167].

Extracellular adenosine levels are tightly regulated by ADA, which is released from endothelial cells and binds to the cell-surface receptor, CD26, to modulate coronary blood flow and minimize adenosine utilization [94,168]. Additionally, A2AR activation improves mitochondrial oxidative stress during ischemic reperfusion by inhibiting complex 1 activity and reducing superoxide generation [169]. The binding of adenosine to A2AR and A2BR induces vasodilation and enhances perfusion. Consequently, pharmacological agents such as dipyridamole increase endogenous adenosine levels to improve myocardial reperfusion and vasodilation [170]. The selective A2A agonist Regadenoson demonstrates superior protection with fewer side effects during ischemic reperfusion [171]. Additionally, the monoclonal antibody Adonis exhibited agonist activity by Gs protein with increased cAMP level and subsequent coronary vasodilation. These findings suggest the presence of spare adenosine receptors that may be therapeutically exploitable in inducible myocardial ischemia [172,173,174,175].

### 4.3. Purinergic Modulation of Inflammatory Response

During ischemia–reperfusion, neutrophil infiltration constitutes a significant contributor to tissue injury. Neutrophil-derived netrin-1 has been demonstrated to act as a cardioprotective signal by activating myeloid-expressed ADORA2B, thereby limiting excessive leukocyte recruitment and subsequent tissue damage [176]. Netrin-1, a neurodevelopmental-linked protein, has emerged as a critical immunomodulator in IR injury [177,178,179,180]. Its interaction with ADORA2B is particularly important, as extracellular adenosine signaling is essential for attenuating hypoxia-driven inflammation [180,181,182,183]. For instance, in a mouse model of MI, pharmacological inhibition of ADORA2B using PSB1115 abolishes the preventive effects mediated by netrin-1, indicating that ADORA2B signaling is essential for netrin-1 driven reduction in infarct size [184]. This protective effect becomes particularly evident during prolonged reperfusion periods or in models of permanent coronary occlusion lasting up to 2 weeks, suggesting that early netrin-1-mediated protection can be sustained over longer durations of reperfusion injury [185]. These findings suggest that targeted deletion of netrin-1 in macrophages results in a reduction in low-density lipoprotein receptor-deficient mice, thereby promoting macrophage emigration from atherosclerotic plaques [186].

Notably, macrophage-specific deletion of netrin-1 confers protection against the development of abdominal aortic aneurysms in mice [187]. The cardioprotective roles of netrin-1 are facilitated through its receptor deleted in colorectal cancer (DCC), which is expressed on endothelial cells and myocytes that activate the ERK1/2-eNOS signaling, leading to enhanced NO production and improved vascular and myocardial function [179,180]. Conversely, signaling through another netrin-1 receptor, UNC5 homolog B, is frequently associated with detrimental inflammatory responses during ischemia–reperfusion injury, underscoring the receptor-specific effects of netrin-1 [188]. Under hypoxic conditions, netrin-1 expression is transcriptionally upregulated by HIF-1α in myeloid cells [189]. Netrin-1 promotes the accumulation of extracellular adenosine, potentially through inhibition of adenosine transporters or adenosine-metabolizing enzymes. The resulting increase in adenosine signaling activates the ADORA2B receptor, thereby attenuating inflammatory responses under hypoxic conditions. During remote ischemic preconditioning (RIPC) of the heart, this protective pathway is further amplified through HIF-dependent upregulation of the anti-inflammatory cytokine interleukin-10 (IL-10) [190]. Moreover, RIPC has been shown to reduce myocardial injury in patients undergoing cardiac interventions, including coronary artery bypass grafting [191,192]. RIPC also exhibits the potential to mitigate acute kidney injury [193] and to improve postoperative cognitive performance in patients undergoing cardiac surgery [194]. Additionally, RIPC has been reported to prevent contrast-induced kidney injury in diabetic patients undergoing coronary artery angiography [195].

## 5. Therapeutic Interventions and Pharmacological Synergy

Therapeutic interventions for acute MI primarily focus on restoring coronary blood flow and nutrient delivery to preserve myocardial energy homeostasis and oxygen supply. Although timely reperfusion is critical for limiting infarct size and preventing progression of heart failure, it paradoxically induces MIRI [196]. The duration of ischemia tolerated before irreversible injury varies across species, largely reflecting differences in collateral blood circulation [197,198]. Ultimately, infarct size is determined by the severity of ischemia, defined as the reduction in blood flow × duration of ischemia, rather than by myocardial metabolic demand alone.

Extensive experimental evidence identifies ischemic preconditioning (IPC)as a fundamental strategy for preventing myocardial injury. Cell surface receptors CD73 and CD39 which facilitate the conversion of adenine nucleotide to adenosine are central to this process. Adenosine serves as a critical stabilized of HIF-1α, which coordinates a transcription response to preserve tissue viability. While pharmacological inhibition of HIF-PHDs has shown efficacy in renal disease and appears safe in clinical settings [52,199,200]. Notably, two multicenter clinical trials (Acute Myocardial Infarction Study of Adenosine (AMISTAD) trials I and II) have shown that early intravenous administration of high doses of adenosine during reperfusion significantly reduces myocardial infarct size [201]. However, the clinical utility of adenosine is limited by its extremely short half-life (1–2 s), pronounced side effects, such as bradycardia and hypotension, and rapid receptor desensitization, thereby restricting its tissue-salvage potential [45].

Recent studies have also identified a RIC, defined as a brief, repeated cycle of ischemic/reperfusion applied to a remote organ or tissues from the heart, rather than coronary reocclusion and reperfusion, reduced infarct size following sustained myocardial ischemia [202,203]. Beyond preserving cardiac functions, RIC confers broad multiorgan protection against I/R injury, with documented efficacy in brain, lungs, liver, kidney, intestine, skin, and myocardium [204]. Among the various RIC modalities, intermittent limb ischemia and nontraumatic peripheral nociception stimulation are the most clinically feasible strategies. Comparative studies indicate that remote preconditioning and post-conditioning provide comparable cardioprotective efficacy, although the optimal dosing regimen of RIC remains to be defined [203,205]. Importantly, the effectiveness of RIC is modulated by multiple confounding factors and is mediated through complex neurohumoral signaling pathways linking the complex signal transduction from the heart to remote organs [206].

Purinergic signaling, particularly through the P2X7R, plays a critical role in post-ischemic cardiac dysfunction by triggering inflammatory signaling via NLRP3/IL-1β driven inflammasome axis and promoting T-lymphocyte mitosis and macrophage activation. In murine models, the antimicrobial peptide cathelicidin has been involved in atherosclerosis through activation of platelets [207], the recruitment of inflammatory monocytes [208], and by acting as a self-antigen [209]. In mice, this peptide has been shown to exacerbate MIRI via TLR4 and P2X7R/NLRP3 inflammasome-dependent mechanism [210]. Beyond its inflammatory role, P2X7R promotes T-lymphocytes mitosis, suppresses apoptosis, and promotes macrophage activators [211]. The *P2X7R* gene exhibits high polymorphism, with at least 11 non-synonymous polymorphisms having been identified in its coding region, several of which result in altered receptor function [212]. Notably, the common loss-of-function SNP rs3751143 is significantly associated with a reduced risk of IHD and ischemic stroke (IS), particularly in smokers [213]. Furthermore, epigenetic regulation by circulating miR-150 has been shown to repress P2X7R expression in cardiomyocytes, offering a potential cardioprotective effect against ischemic injury via modulating the cell death pathways [214]. Additionally, P2X7/NLRP3 signaling axis plays a central role in the pathophysiology of sepsis, coordinating inflammatory responses that may be protective against invading pathogen but become deleterious to host tissues depending on the context, timing, and magnitude of the activation [215]. Sepsis-induced myocardial dysfunction (SIMD), affecting approximately 40–60% of patients with septic shock, is characterized by reversible biventricular dilation, reduced ejection fraction, and impaired contractile reserve [216]. Emerging evidence suggests that pharmacological inhibition of NLRP3 using agents, such as MCC950 and melatonin, confers significant cardioprotective effects in experimental models of sepsis. Collectively, these findings identify the P2X7/NLRP3 axis as a promising therapeutic target and highlight its potential for the development of novel interventions in sepsis-associated cardiac dysfunction [215].

Similarly, P2Y11R is localized in cardiac and endothelial cells and modulates the inflammatory responses following I/R in a paracrine manner. Its stimulation during H_2_O_2_-induced oxidative stress reduces mtROS and cellular damage through the activation of the PKCε signaling pathway [217]. P2Y12, antagonist, such as ticagrelor, attenuate macrophages-mediated inflammation during MI and promotes favorable cardiac remodeling [218].

Beyond direct purinergic modulation, several non-purinergic agents also exert cardioprotective effects against MIRI. For instance, the dipeptidyl peptidase-4 (DPP4) inhibitor, alogliptin, attenuates I/R injury via adenosine receptor- and CREB-dependent signaling [219]. Cilostazol, an antiplatelet agent that inhibits phosphodiesterase-III, increases cAMP levels and activates protein kinase A [220]. It reduces the myocardial infarct size by increasing adenosine levels and increasing the production of NO in the myocardium, subsequently triggering the opening of mitoK_ATP_ channels and a reduction in superoxide production [221]. Similarly, the nucleoside transport inhibitor dipyridamole reduces I/R injury via A1 receptor signaling in animal models ischemia [221]. Resveratrol, a natural polyphenol (trans-3,4′,5-trihydroxystilbene), mitigates cardiac I/R injury by stimulating adenosine production and activating A1 and A3 receptors, as well as NO-mediated signaling. Notably, these protective effects are abolished by antagonists such as L-NAME and SPT [222].

HIF-1α serves as a key regulator of the myocardial response to ischemic injury. Evidence from animal models lacking PHD3 and HL-1 cardiomyocytes demonstrate that the stabilization of HIF-1α promotes the survival of cardiomyocytes by enhancing BNIP3-mediated mitophagy following ischemic events [223,224]. Complementing this transcriptional defense, the K_ATP_ channel acts as a critical metabolic sensor that modulates cardiac energetics to mitigate I/R injury during preconditioning events. The opening of the mitoK_ATP_ channel induces the depolarization of mitochondria and suppresses Ca^2+^ overload during I/R [225]. Experimental evidence is supported by genetic models, including mutations in ABCC9, the gene encoding the cardiac-specific metabolism-sensing component of the K_ATP_ channel, referred as a SUR2A protein [226]. Similarly, Kir6.2-knockout mice subjected to hemodynamic stress, whether from hypertension or transverse aortic constriction, exhibit increased susceptibility to Ca^2+^-dependent maladaptive remodeling, rapidly progressing to congestive heart failure and increased mortality [227,228,229]. Pharmacologically, the modulation of K_ATP_ channels remains a robust therapeutic target in I/R injury. Studies have demonstrated that blocking of K_ATP_ channels exacerbates ischemic damage, while opening K_ATP_ channels is beneficial [230]. Consistent with these mechanisms, K_ATP_ channel openers via agonists such as nicorandil, cromakalim, aprikalim, pinacidil, diazoxide, and others, have been shown to confer potent post-ischemic functional recovery and reduce infarct size. Conversely, K_ATP_ channel blockers like glibenclamide, tolbutamide, and HMR 1098 produce worse ischemic conditions [230]. The opening of the K_ATP_ channel is associated with the activation of AMPK and PKC signaling pathways, promoting increased surface expression of sarcolemmal K_ATP_ channels during hypoxic preconditioning in isolated cardiomyocytes [231]. Specifically, a reduced level of ATP/ADP ratio under metabolic cues promotes the opening of the K_ATP_ channel, leading to a shorter action potential duration and stabilizes the cellular membrane potential, limiting sarcolemmal Ca^2+^ influx. The reduction in Ca^2+^ influx mitigates mitochondrial dysfunction and improves post-ischemic functional recovery. Collectively, these findings establish K_ATP_ channel opener as a promising therapeutic target for IHD, highlighting the central role of metabolic sensing and mitochondrial preservation in myocardial I/R injury [232].

Zinc is an essential cofactor that regulates key signaling kinases, such as PI3K [233], AKT/PKB [234], extracellular signal-regulated kinase (ERK) [235], and glycogen synthase kinase 3β (GSK-3β) [234] involve in prevention of I/R injury. During reperfusion, adenosine A2 receptors facilitate the NO production [236], which mobilizes intracellular zinc to prevent reperfusion injury [237]. Conversely, the loss of zinc via NO is a potential cause of reperfusion injury, and preservation of intracellular zinc by an adenosine A2 receptor agonist prevent reperfusion injury. The adenosine analog NECA has been shown to prevents zinc efflux and markedly increase the mitochondrial zinc at reperfusion [238]. Both NECA and exogenous zinc administered at reperfusion significantly reduce infarct size, demonstrating that intracellular zinc is crucial for regulating cardiac homeostasis supported by A2R agonist. The mechanism associated by this zinc efflux regulated by suppressing the exporter ZnT-1, and induces cytosolic zinc level via NO/PKG-dependent mobilization from intracellular binding sites [239]. This redistribution results in increased mitochondrial zinc levels, subsequently inhibiting complex I activity and suppressing mitochondrial permeability transition pore (mPTP) opening, regulated by phosphorylation of mitochondrial GSK-3β, leads to reduction in infarct size [240,241]. Collectively, these findings indicate that exogenous zinc administration during reperfusion significantly reduce infarct size via A2A receptor, highlighting zinc homeostasis as a viable therapeutic strategy for I/R injury [242].

The resolution of I/R injury also depends on metabolic efficacy of immune cells. Mitochondrial dysfunction impairs macrophage efferocytosis, thereby reducing the production of tissue repair factors. Upon the uptake of apoptotic cells, macrophages are reprogrammed toward a “resolving” phenotype that prevents secondary necrosis, limits inflammatory cytokine production, and enhances the release of reparative mediators. Macrophages also serve as a barrier to bacterial infection through proinflammatory polarization and mobilization of biosynthetic precursors using glycolysis [243]. Elevated glucose utilization is additionally required for alternative macrophage activation, which is initiated by the cytokine interleukin-4 (IL-4) and further accompanied by increased oxidative phosphorylation [244]. Efferocytosis increases long-chain fatty acid content in macrophages, activates the respiratory chain, and promotes an anti-inflammatory response via metabolic intermediates, particularly NAD^+^. IL4 and apoptotic cells may act cooperatively to program macrophages for tissue repair via efferocytosis-induced mitochondrial activation [245]. Excessive mtROS impaired efferocytosis, conversely scavenging mtROS reduces chronic remodeling in preclinical models remains a topic of debate [246], in clinical test.

Given these multifaced mechanisms, several pharmacological strategies are currently under investigation. Selective A2BAR agonists such as BAY 60-6583 and PHD inhibitors are in preclinical or clinical trials for a range of pathological conditions, including sickle cell anemia, kidney disease, Parkinson’s disease, acute lung injury, and various cancers. In addition, various adenosine receptor-targeted agents, such as ticagrelor and clopidogrel, both of which are P2Y12 antagonists, are being evaluated in clinical trials for several health conditions. Drugs that increase extracellular adenosine, such as adenosine analogs, dipyridamole, an ENT inhibitor, or pentostatin, an ADA inhibitor, may also confer cardioprotective benefits. Agents that enhance A2AAR and A2BAR signaling represent additional therapeutic targets, exemplified by regadenoson, a selective A2AAR agonist (Figure 4). Notably, HIFs activation upregulate the expression of CD73, A2AAR, and A2BAR expression, while suppressing ENT and AK expression, optimizing adenosine signaling. HIF stabilizers, particularly PHD inhibitors such as daprodustat, vadadustat, molidustat, and enarodustat, are already approved in various regions including Japan [247], the European Union (EU), the USA [248], and they represent promising therapeutic approaches for targeting the adenosine–HIF axis to treat ischemic and inflammatory diseases.

## 6. Clinical Benefits and Challenges of Targeting Purinergic–Redox–HIF Network

Extracellular adenosine, primarily generated by the sequential degradation of ATP by the ectonucleotidases CD39 and CD73, exerts potent organ-protective effects, particularly via A2B receptors in traumatic hemorrhagic shock (T/HS) [249]. Ischemic conditioning has been identified as a preventive strategy in MIRI, through pre- and post-conditioning followed by I/R. Ischemic preconditioning was first described in 1986. The duration and multiple episodes of ischemia perfusion demonstrated a cumulative effect on myocardial insults and delayed cell death [250], by preventing metabolic deficits and preserving high-energy phosphate production or accelerating the clearance of deleterious catabolites [251]. Adenosine receptors, specifically P1 receptors, are actively involved in preconditioning in I/R. The A2A and A2B subtypes can trigger the inflammatory response and ameliorate I/R damage [250]. The role of A3 receptor remains poorly defined on I/R and needs to be confirmed in MIRI [252]. CD73-derived adenosine inhibits inflammation and fibrosis in TAC-induced heart failure [119]. Recent evidence further suggests sex-specific differences in the CD73/ adenosine axis across multiple tissues, including the brain and liver [253,254]. Notably, the CD73/ adenosine axis is vital for maintaining tissue integrity and facilitating recovery following myocardial injury [114]. Despite its therapeutic potential, the impact of A2BAR agonist, for instance, 5′-nucleotidase on platelet function or bleeding time [255] requires further investigation.

Purinergic receptors are integral components of complex signaling networks, exhibiting reciprocal crosstalk with classical neurotransmitters and non-purinergic modulators. Notably, neutrophil-derived netrin-1 activates myeloid adenosine A2B signaling, conferring cardioprotection in MIRI [256]. Mechanistically, netrin-1 mitigates I/R-induced mitochondrial dysfunction through a NO-dependent mechanism that suppresses Nox4 activation and promotes the recoupling of endothelial NO synthase (eNOS) [179]. These findings underscore the Nox4-uncoupled eNOS-mitochondrial dysfunction axis as a critical therapeutic target [179]. Consistent with this, pharmacological inhibition of Nox2 and Nox4, using a small-molecule inhibitor such as GLX481304, has been shown to reduce mtROS production and improve post-ischemic contractile function following I/R injury in mice [257]. Similarly, the anthocyanin derivative petunidin exerts cardioprotective effects by limiting Nox4-derived ROS, and inhibiting cardiomyocyte apoptosis via Bcl-2 signaling [258]. Metabolic modulators further converge on this pathway. For instance, metformin suppresses Nox4 expression through activation of adenosine 5′-monophosphate-activated protein kinase (AMPK) signaling, which attenuates ROS production. While teneligliptin, a dipeptidyl peptidase-4 (DPP4) inhibitor, exhibits comparable effects by enhancing endogenous glucagon-like peptide-1 (GLP-1) [259]. Collectively, these findings support NOX inhibition and GLP-1 agonist-based therapies as promising strategies for mitigating MIRI.

Translation of these mechanisms into clinical practice is complicated by biological modifiers. Sex-specific differences are evident, with males exhibiting a more proinflammatory phenotype and females demonstrating relative anti-inflammatory protection in inflammatory cardiac conditions such as autoimmune myocarditis [260]. Furthermore, metabolic conditions, such as short-term fasting, reduce HIF-1α levels independent of overt proinflammatory cytokine changes [261], indicating context-specific regulation [262]. Temporal factors are equally important; circadian rhythms significantly modulate HIF-2α activity, contributing to day-time-dependent variation in MI, while the interaction between circadian genes and HIF-1α remains unclear [263]. Additionally, core clock gene, such as PER2, and their interactions with HIFs are poorly defined and need further investigation [95]. Notably, the interaction of BMAL1, a clock gene, with non-canonical partners across various cell types may protect against ischemic injury, potentially enhancing the efficacy of RIPC for treatment of ischemic disease [264]. Beyond MI, circadian modulation of hypoxia and inflammatory responses underscores the “clock-based” therapeutic strategies, offering broader potential for the treatment of ischemia-related pathologies [265].

## 7. Conclusions

Targeting the purinergic adenosine pathway in conjugation with HIF and ROS signaling represents a multifaceted and mechanistically robust strategy to mitigate the I/R injury. The CD73/ adenosine axis exerts pronounced cardioprotective effects by modulating inflammatory responses, limiting oxidative stress, and preserving myocardial function. Concurrently, targeting Nox and downstream ROS signaling effectively prevents mitochondrial dysfunction and cardiomyocyte apoptosis, emerging as a promising therapeutic target. However, successful clinical translation requires careful consideration of key biological modifiers, including sex-specific differences, metabolic state, and circadian regulation, all of which may potentially improve the efficacy of ischemic conditioning strategies and therapeutic outcomes. In addition, critical knowledge gaps remain unclear. The role of purinergic receptor subtypes, such as A3 receptor, as well as their interaction, needs to be investigated. Potential off-target effects of pharmacological agents, together with the intricate crosstalk between pathways and timing of reperfusion interventions, further complicate clinical application. Overall, this review highlights the therapeutic potential of integrating purinergic, HIF, and ROS axis, while emphasizing the need for context-dependent and precision-based approaches. Further, strategies incorporating RIC alongside targeted modulation of these signaling pathways may offer novel and effective therapy to reduce I/R induced injury and improve clinical safety and outcomes.

## Figures and Tables

**Table 1:** Various MI subtypes and key characteristics according to the Fourth Universal Definition of MI (2018).

MI Type	Cardiac Troponin (cTn)	Key Characteristics	Additional Features
**Type 1**	≥1 value above 99th percentile URL	Spontaneous MI due to a primary coronary event (e.g., plaque rupture or erosion)	New ischemic ECG changes; identification of coronary thrombus by angiography or autopsy
**Type 2**	≥1 value above 99th percentile URL	Imbalance between oxygen supply and demand, coronary atherosclerosis, with or without intramural hematoma non-atherosclerotic condition (especially in young women)	Insufficient blood flow to the ischemic myocardium, more frequent in women, associated with higher mortality than Type 1 MI patients
**Type 3**	≥1 value above 99th percentile URL	Sudden cardiac death with symptoms suggestive of MI (before biomarkers available); ST segment elevation in varies from 3% to 24%	Often associated with presumed new ischemic ECG changes or ventricular arrhythmias before biomarker assessment
**Type 4a**	**>5× 99th percentile URL**	MI related to percutaneous coronary intervention (PCI) with, necrosis with or without intra-myocardial hemorrhage	New ischemic ECG changes, imaging evidence of new myocardial loss, or procedural complications
**Type 4b**	**>5× 99th percentile URL**	MI associated with stent thrombosis (confirmed by angiography or autopsy)	Classified using Type 1 MI criteria
**Type 4c**	**>5× 99th percentile URL**	MI related to diffuse restenosis or complex lesion associated with rise and fall of the cTn values above 99th percentile	Classified using Type 1 MI criteria
**Type 5**	**>10× 99th percentile URL**	MI related to coronary artery bypass grafting (CABG)	Development of new pathological Q waves, imaging evidence of new myocardial loss, or angiographic evidence of graft occlusion

## Data Availability

No new data were created or analyzed in this study.

## Abbreviations

The following abbreviations are used in this manuscript:

ADA	Adenosine deaminase
ADORA2A	Adenosine receptor A2A
ADORA2B	Adenosine receptor A2B
AK	Adenosine kinase
AMI	Acute myocardial infarction
AMPK	Adenosine 5′-monophosphate-activated protein kinase
AMISTAD	Acute Myocardial Infarction Study of Adenosine
AREG	Amphiregulin
ASC	Apoptosis-associated speck-like protein
ARs	Adenosine receptors
A3AR	A3 adenosine receptor
A2AR	A2 adenosine receptor
APCP	Adenosine 5′-(α, β-methylene) diphosphate
cAMP	Cyclic adenosine monophosphate
CABG	Coronary artery bypass grafting
CD39	Ectonucleoside triphosphate diphosphohydrolase-1
CD73	Ecto-5′-nucleotidase
CTLA-4	Cytotoxic T-lymphocyte associated protein 4
CAT	Catalase
cTn	Cardiac troponin
Cl-IB-MECA	2-chloro-N6-(3-iodobenzyl) adenosine-5′-N methylcarboxamide
DAMP	Danger-associated molecular pattern
DCC	Deleted in Colorectal Cancer
DMOG	Dimethyloxalyglycine
DPP4	Dipeptidyl peptidase-4
ERK	Extracellular signal-regulated kinases
ETC	Electron transport chain
ENT	Equilibrative nucleoside transporter
eNOS	Endothelial NO synthase
EU	European Union
FAO	Fatty acid oxidation
FIH	Factor-inhibiting HIF
FMN	Flavin mononucleotide
GPCR	G-protein-coupled receptor
GSDMD	Gasdermin D
GRK2	G-protein-coupled receptor kinase 2
GLUT1	Glucose transporter 1
GLP-1	Glucagon-like peptide-1
GSK-3β	Glycogen synthase kinase 3β
HH	Hypobaric hypoxia
HIF	Hypoxia-inducible factor
HIF-PHD	HIF-prolyl hydroxylase
H_2_O_2_	Hydrogen peroxide
HOCL	Hypochlorous acid
HRE	Hypoxia-response element
IHD	Ischemic heart disease
IPC	Ischemic preconditioning
IL-10	interleukin-10
IL-1β	Pro-Interlukin-1β
I/R	Ischemia and reperfusion
IRI	Ischemia–reperfusion injury
IL-4	Interlukin-4
IL-6	Interleukin-6
IS	Ischemic stroke
K_ATP_	ATP-sensitive potassium
MI	Myocardial infarction
MIRI	Myocardial ischemia/reperfusion injury
MAPK	Mitogen-activated protein kinase
mPTPs	Mitochondrial permeability transition pores
mtROS	Mitochondrial reactive oxygen species
NECA	5′-(N-ethylcarboxamido) adenosine
NF-κB	Nuclear factor-κB
NO	Nitric oxide
Nox	NADPH oxidases
O_2_^•−^	Superoxide anion
HO^•^	Hydroxyl radical
ONOO^•^	Peroxynitrite
PCI	Percutaneous coronary intervention
PD-1	Programmed cell death protein 1
PI3K	Phosphoinositide 3-kinase
pVHL	Von Hippel–Lindau
PDK1	Pyruvate dehydrogenase kinase 1
PPARα	Peroxisome proliferator-activated receptor-α
RA	Rheumatoid arthritis
RIC	Remote ischemic conditioning
ROS	Reactive oxygen species
RET	Reverse electron transport
RIPC	Remote ischemic preconditioning
SIMD	Sepsis-induced myocardial dysfunction
SLE	Systemic lupus erythematosus
SOD	Superoxide dismutase
SS	Systemic sclerosis
STEMI	ST segment elevation myocardial infarction
T/HS	Traumatic hemorrhagic shock
TAC	Transverse aortic constriction
URL	Upper reference limit
UTR	Untranslated region
XO	Xanthine oxidase
XOR	Xanthine oxidoreductase
